# Acute myeloid leukaemia with aberrant expression of pancytokeratin: A diagnostic pitfall

**DOI:** 10.1002/jha2.605

**Published:** 2022-11-03

**Authors:** Yasin Dhonye, Haifaa Saadi, Hadil Abu Arqoub, Wael Al‐Qsous

**Affiliations:** ^1^ Department of Pathology Western General Hospital Edinburgh UK; ^2^ Department of Haematology Borders General Hospital Melrose UK

**Keywords:** acute leukaemia, AML, immunohistochemistry, pancytokeratin

1

A 69‐year‐old woman presented with anorexia, arthralgia, fatigue, sweats and palpitations. Her past medical history included an ovarian borderline serous tumour diagnosed in 2018, which was treated with abdominal hysterectomy and omentectomy, and subsequently, following recurrence of a serous carcinoma, with two cycles of carboplatin.

Physical examination showed scattered bruising over upper and lower limbs, but was otherwise unremarkable. Her full blood count revealed pancytopenia: haemoglobin of 93 g/L (115–165 g/L), white blood count (WBC) of 1.7 × 109/L (4–11 × 109/L) and platelets 62 × 10^9^/L (150–400 × 10^9^/L). A peripheral blood film showed circulating blasts. A bone marrow aspirate revealed trilineage dysplasia and an increased population of medium‐large blasts with nuclear folding and prominent nucleoli (10%) (Figure 1). There was also focal loose aggregation that raised the possibility of a non‐haematological malignancy. Immunophenotyping showed the blasts were positive for CD34, HLA‐DR, CD117, CD13, CD33 and CD2. A bone marrow trephine showed trilineage dysplasia with fibrosis and a significant population of medium sized blasts, which accounted for approximately 40%–50% of all nucleated cells (Figure 2). The blasts were positive for CD34 and CD117 with a subset also positive for CD61 (Figure 3). The features were consistent with acute myeloid leukaemia with myelodysplasia‐related changes. An unusual finding was the aberrant expression of pancytokeratin by myeloblasts (perinuclear dot‐like) (Figure 4). The blasts were negative for other epithelial markers including AE1AE3, Cam 5.2, CK7 and CK20. Further investigations revealed loss of chromosome 7 by G‐banding and monosomy 7 by fluorescence in situ hybridisation.

**FIGURE 1:**
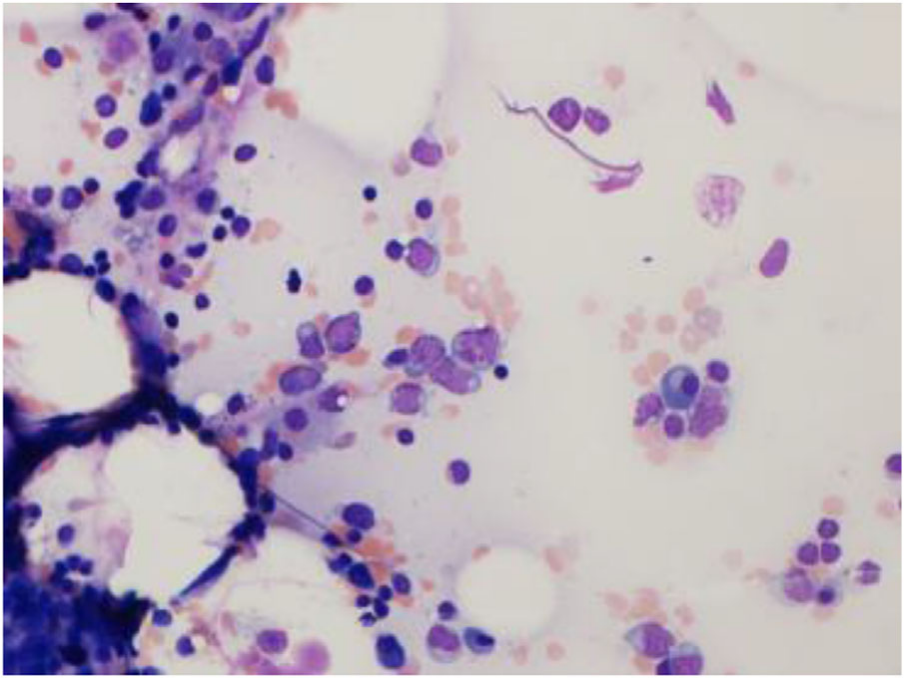
Bone marrow aspirate showing a population of medium to large sized blasts with nuclear folding and prominent nucleoli (Wright–Giemsa stain, 20×)

**FIGURE 2:**
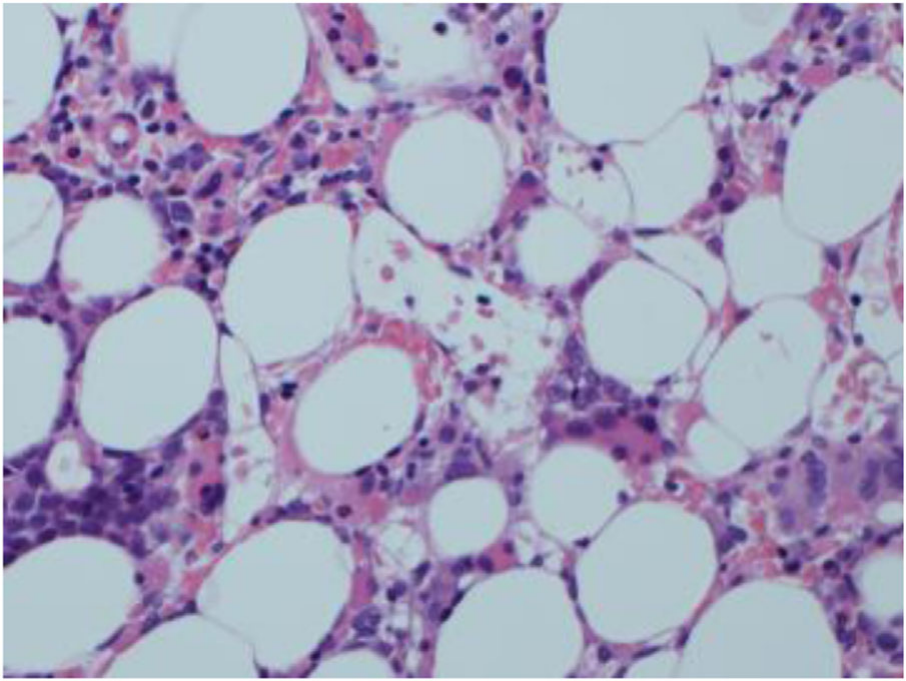
Bone marrow trephine showing trilineage dysplasia and a significant population of medium sized blasts (H&E, 20×)

**FIGURE 3:**
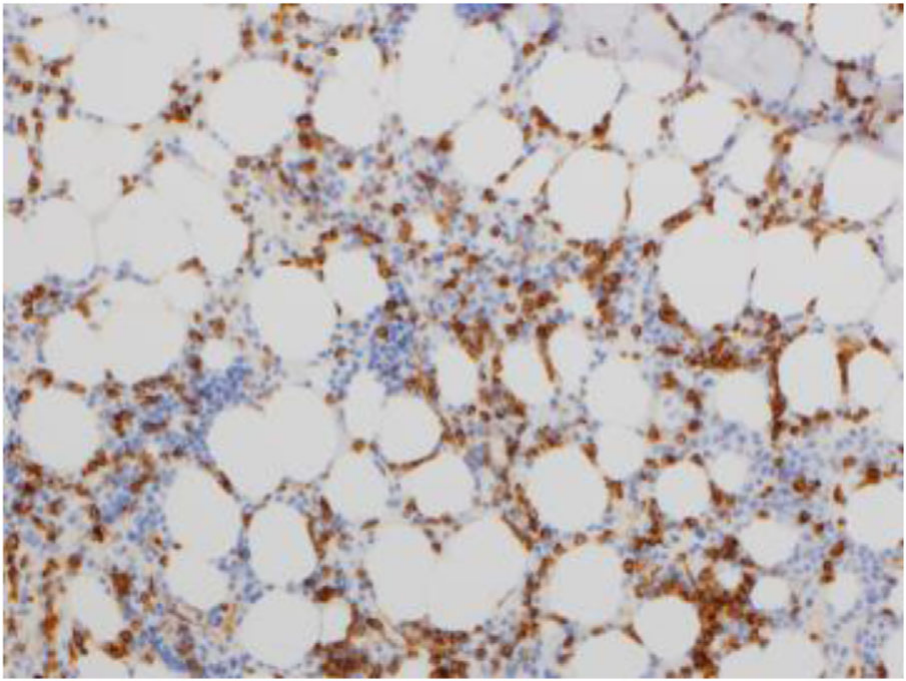
Immunohistochemistry showed the blasts were positive for CD34 (20×)

**FIGURE 4:**
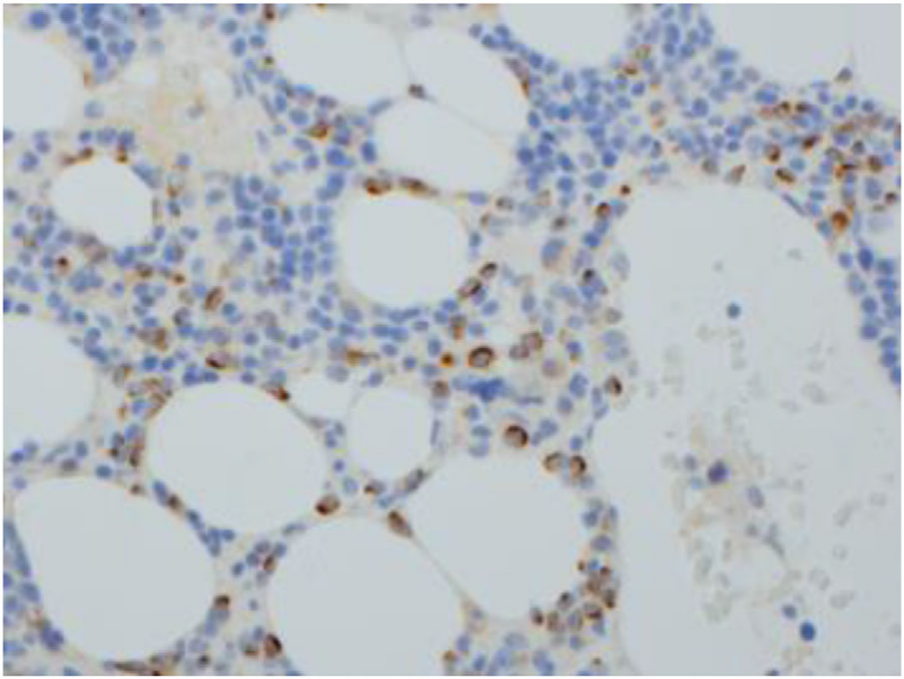
There was aberrant expression of pancytokeratin by myeloblasts with a perinuclear dot‐like positivity (20×)

Aberrant expression of cytokeratins in acute myeloid leukaemia is a recognised but extremely rare phenomenon, which can potentially pose a diagnostic pitfall. Performing a wider panel of immunohistochemistry can help to identify these cases and diagnose them accurately. Our case highlights the importance of interpreting unusual immunohistochemical findings in the context of other investigations including morphology, flow cytometry and the clinical presentation.

## AUTHOR CONTRIBUTIONS

Yasin Dhonye and Wael Al‐Qsous wrote the original manuscript. Haifaa Saadi reviewed the bone marrow aspirate and provided slides for images. Hadil Abu Arqoub and Wael Al‐Qsous reviewed the bone marrow trephine and took images. All authors reviewed and approved the final manuscript.

## CONFLICT OF INTEREST

The authors declare they have no conflicts of interest.

## FUNDING INFORMATION

The authors received no specific funding for this work.

## ETHICS STATEMENT

No research on human was performed on this case. A written informed consent was obtained from the patient.

